# Characteristics of Outbreaks Caused by *Clostridium Perfringens* in Japan

**DOI:** 10.14252/foodsafetyfscj.D-24-00020

**Published:** 2025-09-26

**Authors:** Takahiro Ohnishi

**Affiliations:** National Institute of Health Sciences, 3-25-26 Tonomachi, Kawasaki-ku, Kawasaki-si, Kanagawa 210-9501, Japan

**Keywords:** *Clostridium perfringens*, outbreak, foodborne illness

## Abstract

*Clostridium perfringens* is one of most problematic foodborne bacteria in Japan. The number of outbreaks caused by *C. perfringens* has not decreased. To clarify the characteristics of outbreaks in Japan, data in Annual Statistics of Food Poisoning Japan from 2000 to 2022 published by Ministry of Health, Labour and Welfare, Japan was analyzed.

## Introduction

*Clostridium perfringens* is a gram-positive, spore-forming rod-shaped bacteria. It is present in foods, feces and environment^1^^,^^2^^,^^3^^,^^4^^)^. However, all strains of *C. perfringens* do not cause foodborne illness. *C. perfringens* strains that are related with foodborne illness have the cpe gene that encodes for enterotoxin CPE^5^^)^. Because *C. perfringens* is anaerobic and spore-forming bacteria, spore survive during cooking and vegetative cells of *C. perfringens*, which germinated from spore, is able to grow in the anaerobic condition that is induced by cooking. The growth temperature and generation time of *C. perfringens* is 12 to 50°C and 8 to 10 min, respectively. Therefore, if foods are not cooled rapidly and hold at abuse temperature, *C. perfringens* easily grow to the amount that trigger foodborne illness. Because of these characteristics, it has been supposed that mass-cooked stewed dishes which tend to be anaerobic condition by cooking and were difficult to cool rapidly, were the cause of this illness in Japan. The number of illnesses per outbreak on the foodborne illness caused by *C. perfringens* in Japan has been very large due to eating mass-cooked dishes. *C. perfringens* has been supposed as one of important foodborne microorganisms for long time in Japan^6^^)^. However, there is no tendency to decrease the foodborne illness caused by *C. perfringens* in Japan^6^^)^. In this study, we analyzed the incidence of *C. perfringens* with the data of Annual Statistics of Food Poisoning Japan published by Ministry of Health, Labour and Welfare, Japan^6^^)^.

## Methods

The data regarding foodborne illness were acquired from Annual Statistics of Food Poisoning Japan from 2000 to 2022 published by Ministry of Health, Labour and Welfare, Japan^6^^)^. The number of outbreaks, the number of outbreak-associated illnesses, the average number of illnesses per outbreak, trends in outbreaks and illnesses, venues where outbreaks occurred, and foods linked to outbreaks were analyzed. The figures were generated for manuscript in Microsoft Excel.

## Results and Discussion

**Fig. 1.**
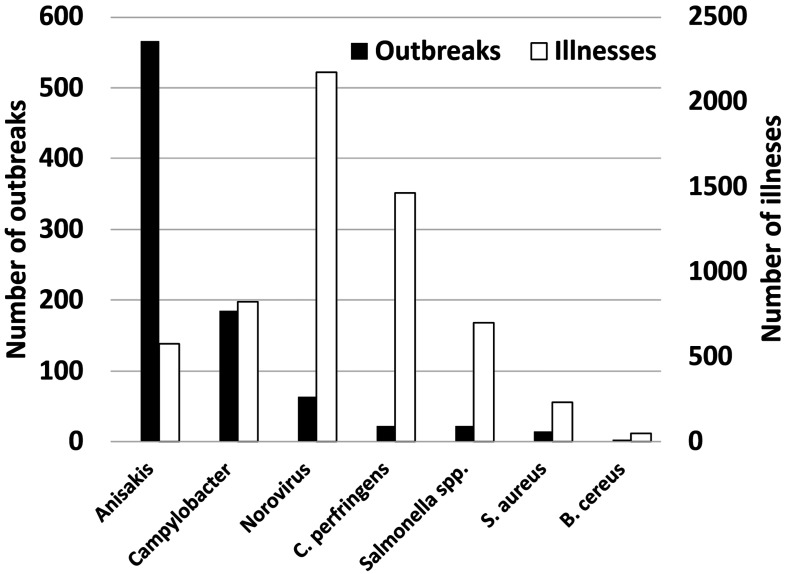
Foodborne outbreaks in 2022

**Foodborne outbreaks in 2022.** Occurrences of foodborne illness in 2022 was totaled (**Fig. 1**). It was *Anisakis* that had the largest number of outbreaks in 2022. Because the number of illnesses per outbreak was almost one, the number of illnesses in Anisakis outbreak was 578. Even though, the number of outbreaks in Norovirus outbreaks was 63, the number of illnesses was 2175 in Norovirus outbreaks. In 2022, there were 22 outbreaks caused by *C. perfringens* and the number of illnesses were 1467. The outbreaks caused by *C. perfringen* was the second largest in the number of illnesses. However, the number of illnesses per outbreak in *C. perfringen* and Norovirus outbreak was 67 and 35, respectively. Therefore, the largest foodborne outbreak regarding the number of illnesses per outbreak was that caused by *C. perfringens*.

**Fig. 2.**
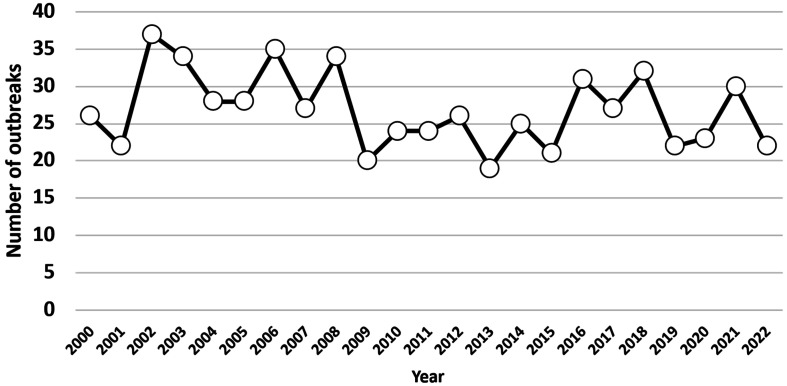
Annual change of outbreaks caused by *C. perfringens*

**Fig. 3.**
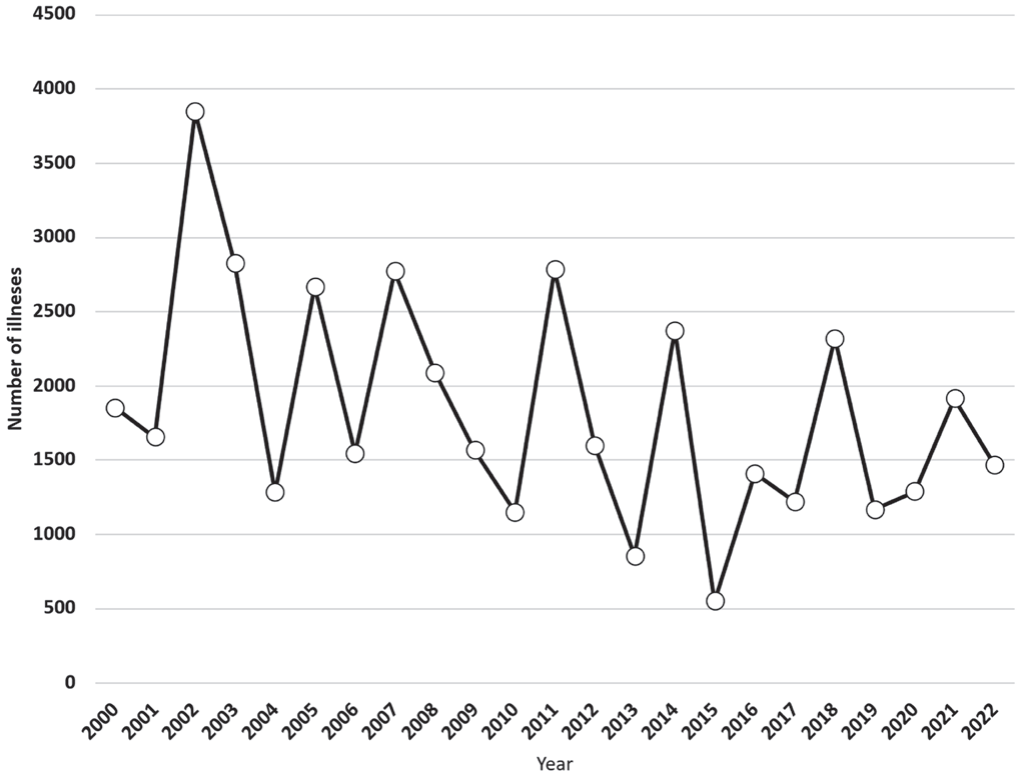
Annual change of number of illness in outbreaks caused by *C. perfringens*

**Annual change of outbreaks caused by *C. perfringens*.** The number of outbreaks caused by *C. perfringens* has been changed by year, roughly from 20 to 30 outbreaks a year (Fig. 2). The number of illnesses was over 1000, excepting in 2013 and 2015 (Fig. 3). There were no changes across the study period in the number of *C. perfringens* outbreaks or outbreak-associated illnesses. These data suggested that it was needed further strategy for prevention of outbreaks caused by *C. perfringens*.

**Fig. 4.**
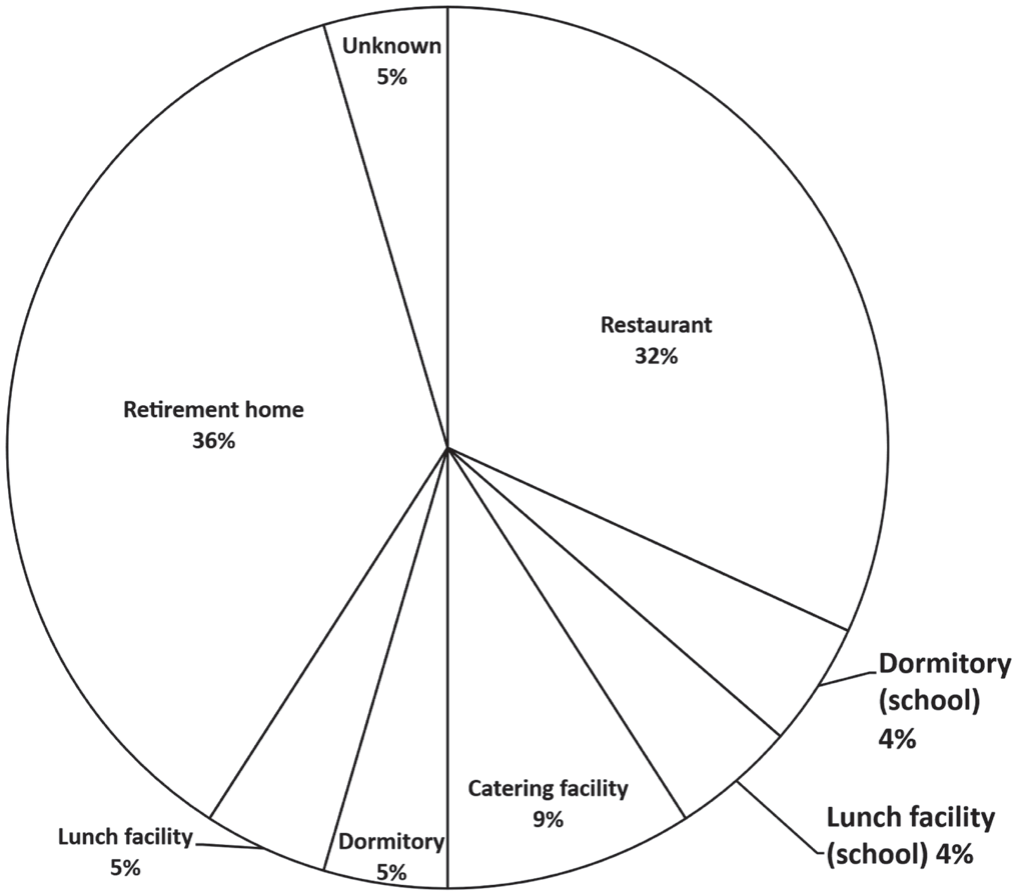
Causative facilities of outbreaks caused by *C. perfringens* in 2022

**Causative facilities.** The outbreaks caused by *C. perfringens* mainly occurred at large cooking facilities in Japan. There were only few cases in homes. In 2022, causative facilities comprised eight retirement homes (36%), seven restaurants (32%), two catering facilities (9%) and other lunch facilities (Fig. 4). The outbreaks occurred at retirement homes have been increased recently. There are a lots of small retirement homes in Japan. In such facilities, staff may not be well educated for hygiene and it may lack enough equipment for mass cooking, such as a chiller for foods. In Japan, manual for hygiene management at large-scale food preparation facilities has been issued by Ministry of Health, Labour and Welfare, and HACCP based food hygiene practice was recommended in this manual. Although the subjects of this manual are large-scale food preparation facilities, small scale facilities should also manage sanitation controls based on HACCP.

**Fig. 5.**
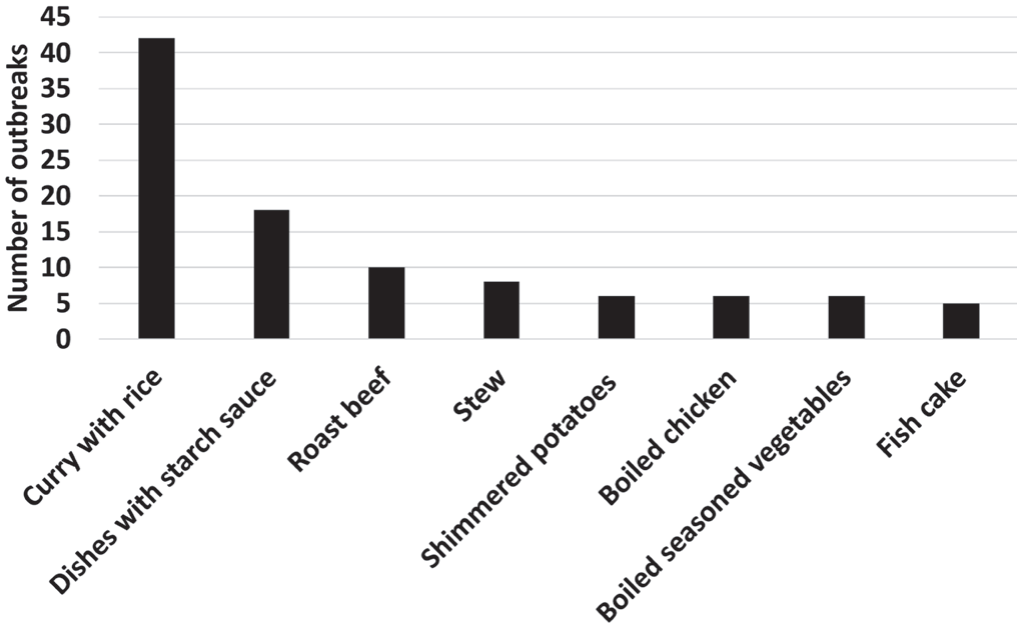
Causative foods of outbreaks caused by *C. perfringens* from 2000 to 2022

**Causative foods of outbreaks caused by *C. perfringens*.** There were 617 outbreaks caused by *C. perfringens* from 2000 to 2022. The causative foods were identified only in 37% cases. There were 42 outbreaks related with curry with rice (Fig. 5). It was the highest number among outbreaks which causative foods were identified. Curry with rice is one of the most popular dishes in Japan, and katsu curry (Japanese curry with cutlet and rice) is now eaten all over the world. In Japanese restaurant, curry is usually prepared in large pot. Cooking large amount of foods results in anaerobic conditions within the food. Furthermore, cooling large quantities of foods are thermodynamically challenging to do quickly. Because of the extended time that the food is within growth range of *C. perfringens*, populations can easily grow to levels that are associated with foodborne illness. There were 18 outbreaks related with the dishes with starch sauce. Because starch sauce has a bit thicker consistency, starch sauce prevents food from cooling and preserves anaerobic condition in food after cooking. Therefore, it is supposed that dishes with starch sauce is good for growth of *C. perfringens*. In most of outbreaks caused by *C. perfringens*, causative foods have not been identified. To efficiently prevent outbreaks caused by *C. perfringens*, it is important to clarify the source of contamination and take measure to deal with outbreaks.
